# Management of unexplained: poly-consultation and hyperfrequency on Primary Health Chilean Attention. A qualitative and exploratory study

**DOI:** 10.1186/s13033-021-00436-4

**Published:** 2021-01-14

**Authors:** Nicolás Fuster Sánchez, Diego Rivera López, Hugo Sir Retamales, Constanza Gómez Pérez, Magdalena Rodríguez Torres

**Affiliations:** Universidad de Valparaiso, Valparaiso, Chile

**Keywords:** Hiperfrequency, Primary health care, Unspecific, Poly-consultant, Discomfort

## Abstract

**Background:**

In Europe, Latin-America, and Asia, poly-consultation has become a complex problem for managing different healthcare systems. However, in the current literature, little attention has been paid to exploring territorial and critical analysis perspectives to manage unexplained symptoms. The purpose of this study is to analyze the socio-structural elements that underlie the users’ phenomenon of poly-consultation or hyperfrequency in the Chilean primary healthcare system (PHCS).

**Methods:**

This paper represents qualitative data collected as part of an exploratory study that used mixed methods across three metropolitan areas of Santiago, Valparaíso, and Concepción, Chile. The study involved a sample of 24 subjects from administrative and management positions in PHC who were recruited from Family Health Care Centers, considering urban municipalities from the low, medium, and high stratum. The study collected data using one set of semi-standardized interviews during a year—data analysis using qualitative content analysis.

**Results:**

This article shows that poly-consultant patients provide a critical clinic category to management that cannot be cover by current biomedical models. Data showed the strain of a somatoform category, especially in the clinic and epistemological exercise. Precisely, the relevance of Chile’s case, a mixed health system, and their effects: the naturalization of collective problems managed as individual problems.

**Conclusions:**

The study results can inform healthcare professionals and managers of developing practical and territorially based. We conclude that hyperfrequency and poly-consultation in Chile reveal relevant stratification in the territory. Those particularities open an opportunity to study quantitative methods, including current analysis categories, to develop new research.

## Introduction

The present article shows the qualitative phase results made in a research project that addresses hyperfrequency in the Chilean primary healthcare system (PHCS). This study's general objective is to understand socio-structural elements that underlie hyperfrequency in metropolitan areas of Chile. The specific ones are: (a) build a social topology of poly-consultant patients, (b) analyze poly-consultation management in PHCS through plans, regulations, and attention protocols, and, (c) describe symbolic construction poly-consultant patients about their daily management affection on the Family Health Centres.

We postulate that poly-consultant constitutes a critical management category. Mainly, prefiguring a social topology that promotes an unspecific symptomatology experience on users that current biomedical models cannot cover. Likewise, we affirm the existence of socio-structural problems that underlie hyperfrequency problems, which will abord critically.

The present article promotes a view that considers administrative and management positions in Public Health to analyze those variables. Precisely we start with a brief account of the recent history of Chilean Healthcare as a context. We propose a discussion for poly-consultation or hyperfrequency implications for a brief background. In that sense, we use qualitative methods for 24 semi-structured interviews, considering urban municipalities from the low, medium, and high stratus of Chilean’s metropolitan areas, according to the National Socioeconomic Characterization Survey. Results came in four major themes and an emergent category. In order: relevant stratification criteria in the territory, social symptoms, multiple symptom management models, temporalities, and diagnosis. The discussion considers specialized literature on somatoform disorders, the strains of hyperfrequency clinic category, a critical problematization of health social determinants, indicators for maintenance of healthcare insurance, and a socio-structural perspective about social relations neoliberal context. Finally, conclusions open possibilities for practical and territory-based manages and quantitative methods.

## A brief account of the recent history of Chilean Healthcare

It is necessary to describe the current Chilean Healthcare system characteristics and their context to highlight its importance, especially with a neoliberal context and management.

In 1979 during Pinochet's dictatorship, twenty-seven health services and the National Healthcare Fund (FONASA, for its name in Spanish *Fondo Nacional de Salud*) were created, trying to decentralize healthcare services into a National Healthcare Services System (SNSS).

Later, in 1980, the Primary Health Care Clinics were transferred to local municipalities, which, according to the World Health Organization (WHO), would have affected healthcare equity, as economic differences in the municipalities’ structures caused inequalities in users’ care. However, the most radical healthcare system reform took place when the Social Security Institutions (ISAPRE, for its initials in Spanish *Instituciones de Salud Previsional*) were created in 1981, leading to a sustained drop in public system financing, from 3.3% in 1974 to less than 2% in 1990 [1, 2]. In 2008, the GDP percentage reserved for public healthcare reached the figures it used to have in 1974. Nevertheless, expenditure has increased significantly, even above OECD countries (7.3% of GDP), but is still far below the average for OECD countries (9.3%), and since 2013 there has been a slowdown.

The mixed provision system has produced significant inequalities between benefits and sustained pressure on the public system. It has had to deal with increasingly scarce resources and universal coverage that forces it to receive significantly higher affiliates. By 2011, more than 80% of Chile" 's inhabitants are FONASA" 's members or beneficiaries, while only 13% are ISAPRE" 's. The diagnosis is even worse if we consider that the private system spends per capita exactly twice as much as FONASA [2].

These numbers must also be comparate with healthcare financing ways. In most OECD countries, public spending on this item exceeds 70% as an average, with Chile and the United States exceptions. Chile barely reaches 49% of public expenditure, a heavy burden on households responsible for financing almost a third of the cost, compared to the average for OECD countries, which barely reaches 20%. This implies the management of healthcare problems with individualization, considering a symbolic and sociological market based (neo)liberalization. Moreover, the number of health professionals is also noticeably low than in other international organizations countries, placing Chile in the last place next to Turkey in the physician’s quantity, with 1.7 per 1000 inhabitants, well below the 3.3 OECD average in 2012 (not even reaching the standard of 2000 which was 2.7). Similarly, in Chile’s nursing professionals case, it reaches 4.2 per 1000 inhabitants in 2012, compared to 8.8 for the organization" 's member countries. These structural pressures and limitations are transferred to Chilean public healthcare professionals, imposing a competitive market model that installs life just as any other commodity [3, 4].

Considering this frame, we propose to problematize poly-consultation or hyperfrequency problems in an exploratory way.

## Poly-consultation or hyperfrequency: a brief background

Poly-consultation, i.e., repeated consultation by the same user to the healthcare services, reaches the clinical category after six months of manifestation according to the ICD-10 and DSM IV diagnostic manuals [5, 6]. It is then called “somatization disorder” or somatomorphic disorder, “as a” group of disorders that include physical symptoms for which no medical explanation has been found, but which are serious enough to cause the patient to have an impairment in work or social functioning" [5].

In Europe, the most commonly used term for these patients is hyperfrequent, while in Chile, the most widely used name is poly-consultant. Despite this differentiation in conceptual terms, when the patient profile enters, it varies depending on the territorial and socio-cultural context. In general, is associate with psychological disorders, such as depression, and others such as childhood trauma or anxiety disorders [5, 6].

Poly-consultation has become a complex problem for the management of different healthcare systems, especially about these ‘models’ economic aspects [5, 7, 8]. In the United Kingdom, somatic symptoms and syndromes represent 20% of primary healthcare consultations. In the Netherlands, the complaint of somatization among outpatients represented 25% of new referrals, while in the United Kingdom, it was 35%. Prevalence rates, and unexplained primary care symptoms from 11 to 60% [9]. In Spain, is estimate that 15% of the population can be poly-consultants. This group consumes approximately 50% of the medical consultations in healthcare centers, responsible for 65% of health costs [10]. As well as the progressive increase in this phenomenon during the 2010–2017 period, mainly in the re-consultations of hyperfrequent users in emergency care [11].

Territorially speaking, a higher prevalence in centers located in rural and smaller areas; however, when considering the hyperfrequent users number per professional as a measure, intermediate size centers are those which present the highest hyperfrequency [12]. Notwithstanding the above, most of the existing research analyzes the hyperfrequency phenomenon only from the’ ‘user’s characteristics. Although other works using multilevel methodology have been able to verify the factors related to the professional and the organization explain, the consultations use variability between 55 and 63% in PHCS [13].

In Latin “America’ s case, Brazil and Chile stand out with common mental disorders, high prevalence rates characterized by the presence of somatomorphic claims [14]. In this regard, several studies highlight the social, psychological, and cultural nature at the base of these disorders and, therefore, the lack of clarity on limits for this phenomenon and its correct classification or definition.

We emphasize this phenomenon of high prevalence in Costa Rica. Accounting estimates consider that 20.8% of the annual healthcare budget allocates to poly-consultation users of ambulatory care centers. Poly-consultation demographic represents 19.75% of the population served in the clinics. This group comprises married women or women living with a partner aged 30–50 with a complete primary education [7]. In Chile, this phenomenon prevalence is exceptionally high (approximately 16%), with Santiago standing out with 17.7%, according to a WHO study [15].

Because of this phenomenon, a problematization is a social object; it is appropriate to include some more general problems. In this direction, our research seeks to understand the socio-structural implications related to meaning construction associated with healthcare within Chile’s primary care network. In this regard, we postulate as a general hypothesis: poly-consultant patients constitute management and mental health models critical category. They are prefiguring a social topology that promotes non-specific and diffuse symptomatological experience. Unfortunately, it cannot be cover by current biomedical models. In this regard, we consider that sociostructural elements are at the root of the discomfort expressed as hyperfrequency. Furthermore, we consider that hyperfrequency management logic reduces PHCS overload without suggesting any change for the discomfort-specific causes. Precisely users’ meanings and experience are symptoms that the current treatments cannot define, and this performs a chance to investigate their causes.

## Methods

The findings presented reports on semi-structured interview data collected as part of a more extensive exploratory study that contemplates mixed methods to analyze the socio-structural elements that underlie the users ‘phenomenon of poly-consultation or hyperfrequency in the Chilean primary health system (PHCS). This study has an epistemological approach of Pierre’ Bourdieu’s [16] “structuralist constructivism” seeking strategies triangulation research [17]. This article synthesizes the field's management and the administrative path that made it possible to produce information for developing a quantitative instrument towards a Chilean poly-consultant profile, which carry out collecting data objective from administrative and management positions in PHC.

In practical terms, we used a semi-structured interview strategy. It consists of a conversation guided by topics proposed by the interviewer while providing high levels of thematic flexibility, allowing an opportunity for new aspects of emerging or those not contemplated in the guideline. Among the different types of existing semi-structured interviews, we use a model that approaches the so-called “semi-standardized interview,” which allows reconstructing the knowledge, opinions, and discourses set that the interviewees have about the topic(s) being studied [18].

The study involved a sample of 24 interview from administrative and management positions in PHC who were recruited from Family Health Care Centers, considering urban municipalities from low, medium and high stratus of Chilean’s metropolitan areas. Precisely, three FHC from Metropolitan Valparaíso (Quebrada Verde, Valparaíso, Aviador Acevedo, Quilpué and Concón, Concón); three from Metropolitan Santiago (Dr. Alessandri, Providencia, Manuel Bustos Huerta, Quilicura and Juan Pablo II, Padre Hurtado) and two from Metropolitan Concepción (Baldomero Lillo, Lota and Santa Sabina, Concepción).

### Sampling

Within the metropolitan areas, the most homogeneous urban municipalities of the high, medium, and low status were selected (one for each stratum), based on current National Socioeconomic Characterization Survey [19] (CASEN in Spanish, 2015) data, as we can see on Table 1. Then, in each one, the Family Health Centers (CESFAM, Spanish for *Centros de Salud Familiar*) with the largest registered population were selected. For this kind of research, it was considered relevant to carry out a non-probabilistic sample. As it is an exploratory study, no sample framework allows us to identify patients from CESFAMs that are Hyperfrequent, trying to develop a poly-consultation profile.

**Table 1:** Source: Own elaboration based on Casen 2015 data

Deciles	I–IV (%)	V–VII (%)	VIII–X
*Metropolitan Valparaíso*			
Valparaíso	48	28	24% (Quebrada Verde’s Cesfam)
Concón	30	27	44% (Concón’s Cesfam)
Quilpué	37	33	29% (Aviador Acevedo’s Cesfam)
*Metropolitan Concepción*			
Lota	45	37	18% (Baldomero Lillo’s Cesfam, Lota Alto)
Concepción	33	29	38% (Santa Sabina’s Cesfam)
*Metropolitan Santiago*			
Providencia	5	13	82% (Dr. Hernán Alessandri’s Cesfam)
Quilicura	42	44	15% (Manuel Bustos Huerta’s Cesfam)
Padre Hurtado	70	26	3% (Juan Pablo II’s Cesfam)

### Analysis technique

The "Qualitative Content Analysis" was used as a mechanism of analysis, understood as:

a technique for interpreting texts, whether they are written, recorded, painted, filmed (…) or in any other form where there may be all kinds of data records, interviews transcriptions, speeches, observation protocols, documents, videos (…). The common denominator of all these materials is their ability to contain material that, read and interpreted accurately, opens the door to various aspects of knowledge and social life phenomena [20].

According to the author, these texts reading should be systematic, objective, and able to be replicated. This analysis technique has the characteristic of mixing observation, data production, interpretation, and content analysis. Interviews were transcribed verbatim, and observational data from field notes were incorporated and analyzed. We considered four aprioristic categories: relevant stratification criteria in the territory, multiple symptom management models, social symptoms, and temporalities (Table 2).

**Table 2:** Source: Own elaboration data matrix analysis

Descriptor	Major themes	Indicators / Subthemes
What do you know	Relevant stratification criteria in the territory Social symptoms	Neglect Precariousness Childcare Inefficiency of derivation Migration Ethnicity Religion and related behaviours Mental health Unexplained symptoms Psychological discomfort Social discomfort
How do you manage	Multiple symptom management models Temporalities	Organizational culture How many medical consultations make someone poly-consultant? Poly-consultant profile Participatory design Medical derivation Human resources Key performance indicators for maintenance of healthcare insurances Digital clinic management Electronic health record Intercultural health care

## Results

### Relevant stratification criteria in the territory

When the investigation began, we decided to seek some socio-structural indicators to determine how territory performs an analysis base.

Excuse me, but we have patients who are known to be poly-consultants, but they are, in general, they are patients with senile dementia or people with their mental disorders as… we know them. A lady comes so much that her daughter, who comes to the doctor's attention, is torn out.

Our study subjects consider socio-structural elements like precariousness, psychological discomfort, or mental health elements to understand hyperfrequency. Nevertheless, their explains come with management elements, according to their positions and sometimes their healthcare expertise.

So the ones from [territory] are different, so in general, people here are like quiet. There are city neighborhoods that we have known all our lives; as I said, the ones that slip through our fingers are from that area where we should work more. Over there, we have to act differently, have a particular family doctor, a team that goes frequently enough. We make different strategies to deal with them.

In terms of management, their knowledge about territories they manage using it as an excuse to avoid elaborating specific protocols of poly-consultation.

We have not any register of poly-consultant patients in the CESFAM(…). We know our people.

### Social symptoms

Administrative and management professionals in PHC have several hypotheses of social symptoms in poly-consultation. We found how particularities about precariousness, neglect, childcare, migration, and ethnicity would perform poly-consultation.

#### Neglect

Social neglect and lack of support network are one of the most characteristic found in the investigation. It is clear to administrative health professionals that there is a possible reason to hyperfrequency in PC, giving us the first step to build a poly-consultant profile.

There is a lack of support network, every user is alone with their environment, and they are lonely. They cannot understand what we said, and they have no one to explain it again, so they feel lonely and come back because they know that we will receive them in our health center.

#### Precariousness and childcare

It should be noted that interviews revealed a relation between precariousness and mental health. Indeed, considering contemporary work to understand how care has changed the social dynamic, women and men without caring for children and the elderly.

Now it is revealed that mental health is essential to people. People recognize that, because it means lousy life quality, it made it challenging to be older. It has to be with many things; today, father and mother need to work, and it makes a total…break in family, between knowledge and relation between fathers, sons, and grandpas.

#### Migration and ethnicity

One of the most critical themes in familiar medicine is an adaptation to territory and their changes, and therefore migration became the main topic of management in Chile. Themes like translation, especially with Haitian Creole, or new religious behaviors emerge as new healthcare management spaces.

We have the most significant number of foreign users and migrants. Venezuelans are predominant, but we confuse them with Chilean because of their appearance; they passed "piola" [Chilean expression for unnoticed] until they speak. The Haitians are notorious because of their skin color. We need to keep an eye on them because mothers claim that some boys are Haitian, forgetting that they are not; they are Chilean.

In addition to migration, ethnicity is preset in some territories considering social composition, and it manages. It is described as "others" discourse but in the space of organization culture, instead of a social problem.

There is a gypsy girl who has a drug problem. A lady… several ladies, who have dementia, come here. We already know them, so at times we take blood pressure measurements to reassure them they are OK. Then we call their children for them to come and pick the ladies up.

### Multiple symptom management models

In our results, management has strategies that depend on territory but always came with the "ghost" of maintenance indicators. When we talk about strategies specifically, medical derivation, human resources, and potential poly-consultants' profile appears.

#### Medical derivation

One of the main topics of the sociology and psychology of health appears on medical-user (patient) relation. This research came in manage terms, considering derivation in medical practice to avoid psychological discomfort or alternative therapies.

One gives them an answer (patient) that generally is incorrect, but you give them an answer, so the patient goes quiet; in some way, it is comfortable. However, in the deep, it is not polite. We are saying something that has "no feet or head" [Chilean expression to incoherences]. She/he, many times, occasionally, say nothing or say things that cannot be real.

My idea, alongside the psychologist who set this up, is to complement this with team interventions such as home visits, family studies, and counseling.

It should be emphasized that medical derivation appears to manage multiple symptoms because they need special attention. Precisely, considering our research in Primary Health Care, administrative and management positions interviewed develop strategies considering social integration and function.

The meaning of how this [hyperfrequency] affects them in their context. A family doctor focuses on watch how this affects their functions every day.

### Temporalities

If we consider poly-consultation a critical category, we need to establish criteria for understanding as a poly-consultant user. In that sense, that research found management criteria based on temporalities to difference hyperfrequency with other affections.

It is simple, with more than seven consultations in six months,' we are on alert. Morbidity control is done with poly-consultant observation for evaluation, and we see if it is referred. If "it is a chronic health problem, "it has taken up by a nurse, and if you have a problem related to mental health or addictions, we refer you to the psychologist or our social worker.

Temporalities criteria perform a stage that administrative and management positions took to manage situations and give patients progress.

If we are responsible for people's progress, we can prevent them from becoming poly-consultants and sick by helping them at the right moments.

### Diagnosis

Last but not least, and the emergent category from this research was the diagnosis. Administrative and management positions of PCR said that clinic exercise has a challenge on unspecific symptomatology. Subsequently, semiology appears in the main stage to manage somatoform symptoms.

Semiology (…) was constructed by disease symptom intelligent identification and acute observation of' patients' body signs (…) in the diagnosis of the disease—"clinical or morbid entities"—one must distinguish "primary or peculiar symptoms from secondary or accidental ones. It is precisely the semiological knowledge that allows us to identify the' disease's different manifestations. For example, signs and symptoms, how to look for them and, how to interpret them, performing clinical semiology.

## Discussion

In general terms, the specialized literature relates non-medically explained symptoms to mental health, more specifically to disorders such as depression and anxiety. At least one-third of patients with somatoform disorders suffer from comorbid anxiety or depressive disorders. In this regard, depression, anxiety, and somatization, with a prevalence rate of 10% each, are identified as the most common mental health disorders in PHCS. These are associated with total functional impairment, increased disability days, and high healthcare costs. Thus, psychometric scales assessing depression, anxiety, and somatization are positively related. This fact would reaffirm the overlap in diagnostic criteria and, consequently, the difficulty of classifying this phenomenon. In India, for example, somatomorphic disorders are conceptualized from CD-10 and DSM IV definitions, known as Functional Somatic Symptoms (FuSS) [9].

Internationally, the most common symptoms found in poly-consultant adults of primary care are fatigue, pain, dizziness, general malaise, gastrointestinal symptoms, abdominal discomfort, diarrhea, and constipation [9]. Studies carried out in Southeast Asian countries analyze at a global level the concepts and mechanisms related to medically unexplained symptoms (from now on MUS) and argue that the psychological nature of somatoform disorders is due to the lack of organic origin diseases to explain them. The latter, seen from the predominant, cross-sectional biomedical viewpoint, displaces somatoform disorders to the realm of agent autonomy, where the only people responsible are the patients who suffer from them.

In this direction, research provides exciting characteristics to the hyperfrequent user profile. For example, the older the patient, the higher the healthcare services use. Also, women between 37 and 75 have a worse subjective perception of their health, so they tend to consume higher. In short, the HF patient is characterized as a middle-aged person with a low-level education who belongs to a nuclear family, lives in the nearest central neighborhood, suffers from a chronic disease, and has a psychic dysfunction. This patient makes an average of 15 consultations a year and frequently uses the previous appointment and the scheduled visit by doctor and nurse [21].

In this sense, these studies indicate that creating a definition and clinical delimitation for those patients with hyperfrequency would directly reduce the overuse of Primary Care (PC) [22]. Also, they suggest that the patient’s type should consider according to whether they have chronic organic or mental pathologies or both. The interventions should be adapted to patient type. Other strategies are those mentioned by Fuertes et al. [10], who infer that one of the points for reducing hyperfrequency lies in incorporating non-presence or telephone consultation modalities (telemedicine).

Concerning the overlap between mental health and poly-consultation, studies in Colombia have estimated that 10% of healthcare costs came from consultation overuse (35% and 45% of work absenteeism are due to mental health problems). The somatization disorder prevalence of almost 40% in hyperfrequenters shows that, although they often appear simultaneously, they are not synonymous, making it necessary to distinguish between both terms and search for another kind of cause beyond the disorder itself. 41% of hyperfrequency cases can be considered chronic diseases, 31% to mental disorders, and 15% to acute and chronic stress. Together, these three factors would explain two-thirds of the real phenomenon. In the same sense, when analyzing the most frequent mental disorders present in PC, it could be inferred that they are under-diagnosed in the clinic. This, if we by contrast patients diagnosed with depression, anxiety, or mixed anxiety-depressive disorder records, 5.6% had been diagnosed with depression, 6.3% with anxiety in the clinical history, and 8.5% with mixed anxiety-depressive disorders [23].

However, after conducting a screening survey, it was revealed that 41.9% of the patients had depression symptoms or established depressive syndrome. Anxiety also occurred as a symptom or syndrome in 13.3% of the cases. On this basis, 55.2% of patients tested positive for mental symptoms or syndromes that had not presented in clinical history [23]. Clara Han [24], relates this as depression from time to time, thinking from a socio-structural perspective.

The low diagnostic capacity is especially important in the mental health field since several studies suggest that patients with anxiety and depression are twice as likely to be poly-consultants. The explanation given by the studies is that anxiety-depressive disorders can generate physical symptoms, and they may affect the health condition’s self-perception. Therefore, random consultations number would increase as a result of this poor self-perception. Therefore, the physician needs to consider research into the mental disorders underlying the reason for consultation [25].

In Chile, research on poly-consultation is scant, and most relate hyperfrequency to somatization disorders. In this matter, the specialized literature estimates that somatic symptoms problem without clinical explanation represents PHC's consultations 15–25%, and up to 70% of this consultation type remains unexplained after being evaluated.

What I discovered, however, was a tense entanglement of municipal politics and health services, made even more acute by the fact that it was a municipal election year. Insecurity, fear, resentment, and frustration circulated among the local mental health workers and municipal health officers, affects in which I too became caught [24].

A study by the World Health Organization (WHO) indicates a 17.7% prevalence of this disorder in primary care consultations [5] in Santiago, Chile. There is no poly-consultant patient homogeneous profile. However, several studies agree that poly-consultant users correspond mostly to women who own a home (without formal paid work), with an average age of 42 years, incomplete primary or average education, married, and with some chronic disease [26].

From the mental health area, national research has addressed the phenomenon as a depression manifestation. This disorder affects 30% of primary health level beneficiaries (7.5% of the general population) [27]. In the very same direction, other studies indicate that poly-consultant patients should be treated not only themselves but also their families, since most of them present some family dysfunction, through a family therapy approach from the mental health perspective [28]. Alternatively, assuming Clara Han's words assuming "life is by a thread" [29].

From the mental health units from the primary care level centers, various intervention proposals have shown high effectiveness, among which are: cognitive-behavioral therapies, psychodynamic therapies, and group therapies. However, a significant number of these patients refuse to be classified as having mental health problems, attributing the responsibility to the doctor, since he or she does not provide an adequate response to their physical affliction. It means that the doctor-patient relationship is damaged, and patients insist on the need for more evaluations and tests [5].

The Brief Family Therapy (BFT) approach, whose main characteristics are to be simple, quick, to generate greater user satisfaction and reduce costs, aims to have patients be able to treat their problems in their own homes through objectives set by themselves. BFT seeks to reduce the symptoms and recover the patient’s autonomous functioning.

With this therapy, the patient stopped using medication and did not request any more complementary examinations, thus saving healthcare costs [30].

The healthcare system provides optimal poly-consultation conditions to be produced and perpetuated since the biomedical approach to providing care, and the Cartesian legacy is still deeply rooted in the given attention. Public health administration does not adequately answer to users' demands by using indicators. Healthcare officials tend to displace the psychosocial component to a secondary concern, producing a rupture between the services offer and poly-consultant population demands, not enabling this problem to diminish [31].

### The strains of a clinical category

The somatoform disorder classification is, precisely, the clinical approach to capture a discomfort refractory to the most common organic diagnoses [6]. Therefore, the "hyperfrequency," somatizers," or "poly-consultants" status of those users who repeatedly come for a short period (usually 6 months) is recognized by its name in diagnostic manuals [5, 6, 26]. However, this attempt to capture the medical device is quite questionable, as it allows neither an adequate conceptualization nor a practice around this kind of disorder.

Usually, the biomedical approach is summoning because of its limitations [7, 27]. This study tries to understand that space where some subjectivity is present [32]. It also assumes these critics and mainly focuses on the unspecific psychosocial circumstances.

In this direction, "professionals limit themselves to referring these patients on several occasions, as having "psychosocial problems," seems more to be a new effort to objectify these' subjects' understanding" [32].

Moreover, from clinical experience, physical signs importance and laboratory findings are emphasized to give importance and credibility to patients with non-specific symptoms and subsequent diagnosis. Here we should ask ourselves why this phenomenon is so disregarded by professionals when it leads to a high social and economic burden. It could be explained broadly for the following reasons: (1) The conditions classification in which people develop these non-specific symptoms is diffuse; (2) psychiatrists do not have sufficient experience to be able to deal with these patients; (3) patients with these symptoms do not seek psychiatric help; and (4) general physicians, as well as specialists, do not refer for psychiatric help [33]. Precisely, it opens an epistemic problem related to the semiology and clinic exercise [34].

There are models used in primary care to address this problem that allow us to detect, recognize, and manage non-specific symptoms. Still, the literature highlights the importance of developing customized models that meet each center's needs due to their complex and multifactorial nature. Therefore, an exploratory model is proposed for non-psychiatric users in six specific circumstances, each with its considerations when dealing with these users [33].

Based on this, and around a semiological problem, we find an epistemological problem. Specifically, between biomedicine targeting the disease and the condition experienced by the patient:

Sometimes the plaque that makes the body visible shows the doctor that the patient ‘has nothing’ wrong, contrary to what the latter claims, who complains of various aches and pains. The opposition between the’doctor’s illness and the’patient’s condition is therefore clear. The objective illness proof was not provided (…), so the pain is imputed to the’patient’s sickly fantasy. He is an imaginary patient [35].

As Canguilhem points out, instability and irregularity are (…) the vital phenomena essential characteristics, so forcing them into the metric relations rigid framework means denaturing them [36].

While observing interpretative frameworks that make the pathologies exclusively physiomechanical, physical, or biochemical problems, the biomedical view fails. These, specifically to reveal the seriousness that the body's interaction with its environment would have in the' disease's etiology production.

Given this, life is not indifferent to the material and symbolic conditions that make it possible; hence, it is normative activity [36]. In other words, life produces installing normative standards that allow it to adapt to a continually changing environment. It is why health manifests itself when the pathological, and the associated pain, impedes the’individual’s daily life development. The body, then, reveals its health or illness concerning the resistance and adaptation capacity towards the conditions of an environment that forces it to displace its limits. Those processes would explain the proposals for the biomedical model to be modified at different levels, from the bio-psycho-social approach development to the so-called integrative medicine [37–42].

Problematise the disease and the experience performs an alternative path to research somatoform symptoms, especially if we consider its association with non-specific psychosocial factors.

### Social determinants: a critical problematization

Approaches to social determinants proposed by the WHO [15], could be made in a critical path. We consider that this perspective lacks criticism and specificity of the social environment, avoiding topics like how individualizing biomedical features regulates its incorporation [3].

This issue is crucial and related to scientific medicine's social position and its link to the State and the market [43, 44]. It is not surprising then that both specialized literature and health institutions recognize a biomedical approach's relative incapacity to face the new challenges in healthcare successfully. We consider that this is directly related to how biomedicine is in society as a technique for managing discomfort, namely: processing social problems on an individual basis.

### Indicators for maintenance of healthcare insurances: the dictatorship of numbers

The problem that affects care, diagnosis, and treatment of somatization disorders, must be considered with questions about maintaining and managing neoliberal healthcare.

The literature reports that such benefits generate economic disadvantages with a full agreement-level public care system that is more expensive burdensome, reaching an international prevalence in primary care around 15–22% [7, 26]. This global observation is particularly sensitive to Chile due to the conditions of its public health system.

Within this framework, extreme resource rationalization and optimization [1] imposes that the recognized burdens that hyperfrequent patients represent for the health system take on particular relevance. Studies said that 30% of users consume 80% of healthcare resources. This is a critical number, considering that unexplained somatic symptoms problem fluctuates around 15–25% of total consultations in Primary Health Care (PHC). We consider that it would imply a broad impact of these conditions in the full benefits of primary healthcare services, constituting between 60 and 80% of users' total demand who attend to those centers [26].

Based on international studies, users characterized as hyperfrequent cause twice the cost of any other patient type due to the number of examinations requested and medicines. Considering context, specialized literature agrees on the need to improve the resolution of this sort of disorders, as well as the need to discuss the allopathic, orthodox, scientific, or biomedical medicine limitations [5, 6, 8, 26, 32]. In this direction, it is necessary to have a profile that allows us to know who these hyperfrequent patients are beyond the country's limited case studies. A poly-consultant general profile would enable us to rethink, among other things, the management mechanisms of these patients in the public healthcare system.

### A way of understanding social relations: collective problems managed as individual problems

A fourth problem overlapping with the previous ones has deserved -and still deserves- an in-depth study that shows its importance in the more or less global matrix of assessment deployment that makes up the connectionist world [45]. In this regard, the socio-economic transformations that Chile has undergone in the last 40 years have produced a particular way of understanding social relations [46]. This series of reforms contains a new fiscal discipline, macroeconomic stabilization, market liberalization, state enterprises, and social services privatization. Furthermore, the reduction of State intervention in capital markets and the economy in general [46–48], would be at the base of a new institutionality.

The performance of a (neo)liberal revolution is related to generalized privatization that allows the action of free agents as a base element for the management of their lives [3, 49, 50]. The first result of Chile's transformations is that, in less than two decades, went from being "a closed economy with a high level of state intervention to being one of the most, if not the most, open and market-based economies in the world" [46], whose main support point is individual modulation. Healthcare is not far from these changes, so FONASA's became a critical example of the process.

Since the 1990s, Chilean governments have not (yet) substantially amended any of these reforms, keeping in line with world trends. On the contrary, the country intensified possibility conditions for individuals exposed to available habitat modification. People also could assume social order as natural, and: renewed society, freedom, political conceptions imposed by the weight of the immutable would appear as utopian. Indeed, modify the public administration's logic and committing individuals to manage their lives based on their projects, quite far from community logic [47, 49].

In this direction, we are currently witnessing a highly individual way of experiencing inter-subjective relations [51], which has made possible the transition to a new way of understanding the overlap between State, market, and society, and the role that each of these has in the management of social problems. Generalized privatization of social affairs emerges in which freedom of agents operates to manage their environment and their lives, thus transferring the structural inequality burden. Society design demands citizens who must individually manage their precariousness and their lack of certainties [29, 52, 53].

Crosses over the possible answers to previous points, namely the burden imposed by non-specific somatic consultation and biomedicine's limits in this regard came from the socio-structural perspective. It also conditions the understanding that individuals themselves have of their discomforts and how they deal with them. It is precisely at the intersection of these problems that our research was.

## Conclusions

Multiple choices for research appear to hyperfrequent patients. Firstly, a sociodemographic characterization. Secondly, a problematization about how to care managing emphasizes the governmental rationalities behind the logic of care plans and programs expressed in specific protocols. Thirdly, what obstacles and possibilities people face in a deeply individualized healthcare system such as the Chilean one, considering the symbolic construction of their affections.

As a brief overview, during this article, we have outlined elements that allow us to understand how the Chilean healthcare system manages hyper-frequency from the words of those who execute and handle the medical protocols. In this regard, this research allowed us to find four major analytical categories to understand hyper-frequency in Chile that will structure the study's quantitative phase.

A survey could be a way to investigate poly-consultation considering socio-structural dimensions, looking for space particularities. In that sense, search for multiple symptom management models will allow us to understand how territorial particularities become administration methods that may or may not replicate. Undoubtedly, it is worth thinking whether the social symptoms suggested by the literature on hyperfrequency such as mental health or chronic diseases or abandonment replicates in Chilean.

## Data Availability

The datasets used and/or analysed during the current study are available from the corresponding author on reasonable request.
